# Granulomatosis with Poliangeitis (Wegener’s Granulomatosis): Orofacial Manifestations. Systematic Review and Case Report

**DOI:** 10.3290/j.ohpd.a45433

**Published:** 2020-10-27

**Authors:** María Apoita-Sanz, Patricia Blanco-Jauset, Carlos Polis-Yanes, Rosa Maria Penin-Mosquera, Gallego Montserrat-Gomá, Laura Pozuelo-Arquimbau, August Vidal-Bell, Carlos Arranz-Obispo, José López-López

**Affiliations:** a Dentist, Faculty of Medicine and Health Sciences, Dental Hospital, University of Barcelona IDIBELL, Barcelona, Spain. Bibliographic review, reviewed manuscript, validated final version.; b Dentist, Faculty of Medicine and Health Sciences, Dental Hospital, University of Barcelona IDIBELL, Barcelona, Spain. Dental management, reviewed manuscript, validated final version.; c Dentist, Faculty of Medicine and Health Sciences, Dental Hospital, University of Barcelona IDIBELL, Barcelona, Spain. Bibliographic review, reviewed manuscript.; d MD, Pathological Anatomy Department, Bellvitge Universitary Hospital, Barcelona, Spain. Assessment of the histological samples, reviewed manuscript.; e MD, Pathological Anatomy Department, Bellvitge Universitary Hospital, Barcelona, Spain. Assessment of the histological samples, reviewed manuscript.; f Resident Physician, Maxillofacial Surgery Department, Bellvitge Universitary Hospital, Barcelona, Spain. Medical-surgical management, reviewed manuscript.; g MD, Pathological Anatomy Department, Bellvitge Universitary Hospital, Barcelona, Spain. Assessment of the histological samples, reviewed manuscript.; h MD, Faculty of Medicine and Health Sciences, Dental Hospital, University of Barcelona IDIBELL, Barcelona, Spain; Maxilofacial Surgery Department, Bellvitge Universitary Hospital, Barcelona, Spain. Medical-surgical management, reviewed manuscript.; i Dentist and MD, Faculty of Medicine and Health Sciences, Dental Hospital, University of Barcelona IDIBELL, Barcelona, Spain. Bibliographic review, dental management, reviewed manuscript, validated final version.

**Keywords:** granulomatosis, polyangiiitis, Wegener’s granulomatosis

## Abstract

**Purpose:**

To present an update the orofacial manifestations of granulomatosis with polyangiitis (GPA) and present a clinical case with the initial signs in the oral cavity.

**Materials and Methods:**

A bibliographic search was performed on Pubmed with the keywords ‘Wegener’s granulomatosis’, ‘etiology’, ‘oral manifestations’, ‘oral cavity’, ‘gingiva’. The inclusion criteria were papers published in English in the last 10 years that made reference to clinical cases with in which the oral cavity was affected. The quality of the results was assessed with ‘The 2013 Care Checklist’.

**Results:**

Nineteen clinical cases were analysed. The average quality was 7.68/13 (range 5-10/13). 73.7% of patients were women, the most frequent area for the lesions was the gingiva and the most prevalent lesion was gingival hyperplasia. 68.4% of the patients had this lesion as a first sign, 21.1% as a progression and 10.5% as a recurrence. 68.4% of the lesions resolved once medical treatment was established.

**Conclusion:**

GPA is a multisystem disorder associated with considerable morbidity and mortality if not treated. Early diagnosis improves the prognosis. The first manifestation of the disease can be seen in the oral cavity. It is important that dentists recognise the oral manifestation in order to improve the prognosis.

Wegener’s granulomatosis (WG) or granulomatosis with polyangiitis (GPA) is a rare systemic disease that can be fatal. It is frequently a chronic disease, with periods of remission and relapses. Its etiology is unknown but it is believed to be mediated by the immune system. This disease is included among the forms of vasculitis that affect small- and medium-caliber vessels, and is associated with anti-neutrophil cytoplasmic antibodies (c-ANCA).^12^,^37^ It is pathologically characterised by an inflammatory reaction of the vessel (vasculitis) that can damage the vital organs.^12^ It occurs as a basic triad: necrosis, granulomatous inflammation and vasculitis.^12^,^37^ It especially affects the upper (paranasal sinuses, nose and trachea) and lower respiratory tract (lungs) and kidneys.^12^,^16^

The disease was described for the first time in 1936; in 1954, it was named Wegener’s granulomatosis and in 2012 the disease was renamed as granulomatosis with polyangiitis.^8^,^10^,^18^,^22^

GPA can affect multiple systems, although it is frequently limited to a small number of organs. Less frequently than the respiratory tract and kidneys, it can also affect the skin, central nervous system, heart, salivary glands, eyes and orbits, gastrointestinal tract, spleen, pituitary gland, thyroid and urogenital tract.^1^

This disease occurs with a large and variable range of initial symptoms. These may include fever, general discomfort, headaches, night sweats, anorexia, weight loss, rhinorrhea, chronic epiphora, pus-like or bloody drainage from the nose, nasal and oral ulcers, chronic sinusitis, throat pain, pain and loss of hearing, aphonia, vision loss, dyspnea, salivary glands enlargement, arthritis, arthralgia, myalgia, peripheral neuropathy, abdominal pain, bloody diarrhea and vaginal bleeding.^1^,^14^

## Epidemiology

GPA does occurs equally in both sexes. Its world incidence is not precisely known,^22^ but some authors^1^ report 23.7 to 156.5 cases per million and an annual incidence of 3.0 to 14.4 cases per million. The US has been reported to have 3 cases per 100,000 habitants,^22^, while in Europe, the incidence is 5 cases per 100,000 inhabitants.^12^ GPA can appear in any ethnicity, but it is prevalent in Caucasians. A wide age range is affected (8-99 years old), with an incidence peak between 20 and 40 years old, making the average age of diagnosis between 40 and 55 years.^12^,^22^,^39^

## Etiology

Despite the fact that the etiology of GPA is unknown, anti-neutrophil cytoplasmic antibodies (c-ANCA) are highly sensitive and specific for this disease, suggesting a pathogenic role. ANCA directed against proteinase 3 (PR3) and myeloperoxidase (MPO) have the ability to activate neutrophils, increasing their ability to adhere to the endothelial cells, which in turn induces oxygen release and lithic enzymes that damage blood vessel walls, producing vasculitis.^26^ Recent studies, eg by Morgan et al,^25^ have observed a decrease in the number and function of T lymphocytes, particularly the subpopulation CD4+CD25+FOXP3+, in patients with GPA. On the other hand, the roles of B lymphocytes as an IgG producer and anti PR3-ANCA seem to be clearer, since these cells are frequently found in inflammatory granulomatosis. Chang et al^4^ evaluated the presence of IgG4+ in patients with GPA, comparing it with healthy patients, finding that IgG4 was increased in 18.6% of the samples taken from any location and in 38.1% of the samples taken from the head and neck. Finally, environmental factors along with infections (chiefly *Staphylococcus aureus* and *Cytomegalovirus*) can be considered GPA triggers, seeing that ANCA is not detected in an estimated 25%–50% of patients with GPA.^26^

## Clinical Symptoms, Diagnosis and Histopathology

GPA can be classified according to its severity (Table 1a)^1^,^39^ and the frequency of the various presentations. Some studies state that 3.9%-22% of the patients have a localised form of presentation at the time of diagnosis.^25^ In a study performed in 180 patients by Stone et al,^35^ 71% of the sample had a severe form vs 29% who had a localised form.

**Table 1:** Classification of Wegener’s granulomatosis according to Almouhawis et al^1^ and Wang et al;^39^ diagnostic criteria established by the American College of Rheumatology in 1990^21^

Classification
Localised: when only the upper and/or lower respiratory tract is affected without any other systemic involvement or constitutional symptoms
Early involvement: affects any organ, except the renal system or any other vital organ
Generalised: renal disease or other involvement that entails creatinine levels less than 5.6 mg/dl
Severe: Renal failure or failure in any other organ with serum creatinine levels higher than 5.6 mg/dl
Refractory: progressive disease unresponsive to glucocorticoids and/or cyclophosphamide
Diagnostic Criteria
Ulcerous lesions in the oral mucosa or bleeding or nasal tumefaction
Presence of nodules, cavities or infiltrates in the thoracic radiograph
Alterations in the urinary sediment and hematuria
Presence of granulomatous inflammation on the biopsy

It can take 4.7 to 15 months to make a diagnosis from the appearance to the first symptoms.^14^ For the clinical diagnosis of this disease, the patient must meet two or more of the diagnostic criteria established by the American College of Rheumatology in 1990^21^ (Table 1b). Important aspects in GPA diagnosis are the anti-neutrophil cytoplasmic antibodies (c-ANCA), because these are considered markers of the disease.^22^ Thus, GPA diagnosis is based on the combination of clinical manifestation, ANCA positivity (although up to 20%-50% is negative) and histologic evidence of vasculitis, necrotising glomerulonephritis or granulomatous inflammation of biopsies taken from skin, lungs or kidneys.^12^ However, early diagnosis of the disease is complicated due to its unspecific signs and symptoms, which can mimic other pathologies.^1^ Differential diagnosis is rheumatoid vasculitis, hematological neoplasia such as lymphoma and leukemia, other granulomatous diseases such as sarcoidosis and Crohn’s disease, granulomatous infectious processes (e.g. tuberculosis), foreign body reactions and fungal infections, drug-induced gingival enlargement, pyoderma gangrenosum and secondary lesions due to immunodeficiency.^12^,^34^

Its histopathology is characterised by diverse patterns: necrosis, granulomatous inflammation, vasculitis and immunological deposits. Necrotic areas are irregularly distributed and surrounded by multinucleated giant cells. Granulomatous inflammation is characterised by the presence of macrophages, multinucleated giant cells and other inflammatory cells. Vasculitis shows fibrinoid necrosis that affects vessel walls with an acute or chronic inflammatory infiltrate and occasionally a granulomatous inflammation inside the vessel wall.^1^,^23^

## Treatment

Wegener’s granulomatosis or granulomatosis with polyangiitis (GPA) always requires systemic treatment due to the rapid progression of the disease. The type of treatment depends on the severity of the disease and whether it is localised or generalised.^1^,^23^ When the disease develops rapidly with progressive glomerulonephritis, progressive alveolar hemorrhage and multiple mononeuritis, more aggressive treatment is indicated. It has been suggested to administer flexible therapies according to disease activity, morphologic changes, indications and contraindications of the treatments.^1^ Prior to the introduction of immunosuppressive therapy, the prognosis was poor. Today, the treatment of choice is cyclophosphamide, which acts as an immunosuppressive, combined with corticoids (anti-inflammatory effect) administered orally or intravenously.^34^ Other medications used include methotrexate associated with corticoids, azathioprine, leflunomide, co-trimoxazole, mycophenolate, cyclosporine or rituximab.^1^,^12^

## Dental Management

Dental treatment in such patients must focus on the identification of infections and their elimination. It is expeditious to perform any necessary dental treatment before the start of the immunosuppressive therapy, although it is also true that delaying GPA treatment could be fatal. Hence, when dental treatment is needed, the same recommendations used for patients under immunosuppressive therapy must be followed. During the acute phases, only emergency treatment must be performed and always under previous consultation with the treating physician; daily antibacterial mouthwashes must also be prescribed. During remission or maintenance therapy, interdisciplinary rconsultations, oral prophylaxis including antibacterial mouthwashes, and treatment of any new dental problems must be carried out. Before any invasive dental treatment, antibiotic prophylaxis must be implemented. Nephrotoxic drugs must be avoided. It is necessary to have and analyse the relevant blood test and monitor blood pressure during the interventions.^27^

## Progonosis

Non-treated GPA has a fatal outcome that can be triggered by secondary kidney failure due to the previous kidney impairment. It is estimated that 82% of non-treated patients die after one year. Although immunosuppressive therapy has improved the prognosis of GPA (5-year survival rate: 70%-80%), complete remission is only achieved in 75% of the patients. Recurrence is known in localised and generalised forms and is associated with the formation of granulomas.^1^,^12^

## Purpose

Since primary disease manifestation may first be seen in the oral cavity and because its prognosis depends on rapid intervention, we believe it is justified to provide a literature update on GPA oral and facial manifestations. Thus, the objectives of this review are to present 1. a literature review of the diverse orofacial manifestations related to GPA and 2. a clinical case of a patient in which this disease manifested primarily in the oral cavity.

## Materials and Methods

This review was performed in accordance with the PRISMA guidelines,^24^ complying with 21 of 27 items.

### Search Strategies

A literature search was performed on Pubmed database using the following key words: (‘Wegener’s granulomatosis’) and (‘oral cavity’); (‘Wegener’s granulomatosis’) and (‘oral manifestations’); (‘Wegener’s granulomatosis’) and (‘etiology’); (‘Wegener’s granulomatosis’) and (‘gingiva’).

### Inclusion and Exclusion Criteria

The inclusion criteria to select papers were: published in the last 10 years (the first search was carried out in June 2018 and the last in December 2019 with no new results), articles in English, controlled clinical trials, case series, reviews and clinical cases reports that described oral and/or facial manifestation of Wegener’s granulomatosis. Systematic reviews were excluded for the specific analysis. Finally, since no clinical trials were found, only clinical case reports were used for assessment in this review.

### Study Selection

After performing the electronic search, the titles and abstracts found were read independently by each author. The articles that seem to comply with the inclusion criteria and those that raised doubts were examined in full text and discussed among the authors.

### Study Variables 

Data were collected on age, gender, GPA form of manifestation (oral or facial) and location, whether it presented as a first manifestation, recurrence or progression of the disease, average time until oral lesion re-appearance when it is not the first manifestation, associated symptoms, diagnostic tests, whether biopsy was performed on oral lesions and the result, whether biopsy was performed on other sites and the result, treatment, and whether oral and/or facial lesions were resolved after treatment.

### Risk of Bias 

For quality assessment of the clinical case reports included in this review, the 2013 Care Check List^30^ was used. In this list, 13 items are assessed, with possible scores ranging from zero to thirteen.

## Results

### Literature Search

A total of 280 articles were found. After applying the inclusion criteria, eliminating duplicates and reading the titles and abstracts, a total of 20 clinical case reports were included in the review. No clinical trials or case series were found.

### Risk of Bias

Regarding the quality of the included papers, 2^16^,^29^ (10%) obtained a score of 10/13 points; 5^11^,^14^,^32^,^36^,^38^ (20%) had a score of 9/13 points; 4^5^,^20^,^32^,^40^ (25%) 8/13 points, 2^3^,^33^(10%) 7/13 points; 5^2^,^9^,^13^,^38^,^39^ (25%) a score of 6/13; 2^19^,^36^ (10%) 5/13 points. The average score of all the report cases was 7.6/13 points, with a range of 5/13 to 10/13. None of the articles obtained the maximum score. The studies by Hérnandez et al^16^ and Reboll-Ferrer et al^29^ had the highest score (10/13), and the reports by Candau et al^2^ and Heera et al^15^ had the lowest score (5/13) (Table 2).

**Table 2:** Quality assessment applying the CARE guidelines for case reports: explanation and elaboration document^30^

Author (year)	Title	Key words	Abstract	Introduction	PI	Clinical findings	Timeline	DG	TI	Follow-up	Discussion	PP	IC	Total
Hérnandez et al (2008)	1	1	1	1	0	1	1	1	1	1	1	0	0	10
Hanisch et al (2017)	1	1	1	1	0	1	1	1	1	0	1	0	0	9
Wang et al (2018)	0	1	0	0	0	1	0	1	1	1	1	0	0	6
Fonseca et al (2017)	0	1	0	1	0	1	0	1	1	0	1	0	0	6
Sung et al (2015)	1	1	1	1	0	1	0	1	1	1	1	0	0	9
Staines et al (2009)	0	0	0	1	0	1	1	1	1	1	1	0	0	7
Candau et al (2014)	1	0	0	1	0	0	1	1	1	1	0	0	0	6
Siar et al (2011)	1	1	1	1	0	1	1	0	1	1	1	0	0	9
Ruokonen et al (2009)	0	1	1	1	0	1	1	1	1	0	1	0	0	8
Carter et al (2008)	1	0	1	1	0	1	0	1	1	0	1	0	0	7
Xing et al (2011)	1	0	1	1	0	1	0	1	1	1	1	0	0	8
Ceylan et al (2013)	0	1	1	1	0	1	0	1	1	1	1	0	0	8
Kenis et al (2013)	0	1	0	0	0	1	1	1	1	0	0	0	0	5
Green et al (2013)	1	1	0	0	0	1	0	1	1	0	1	0	0	6
Kosalka et al (2014)	1	1	1	1	0	1	0	1	1	0	1	0	0	8
Reboll-Ferrer et al (2010)	1	1	1	1	0	1	1	1	1	1	1	0	0	10
Ujjawal et al (2016)	1	0	0	0	0	1	0	1	1	1	1	0	0	6
Peters et al (2018)	1	1	1	1	0	1	0	1	1	1	1	0	0	9
Gómez-Torres et al (2013)	1	1	1	1	0	1	1	1	1	0	1	0	0	9
Heera et al (2012)	0	1	0	0	0	1	0	1	1	0	1	0	0	5

PI: patient information; DG: diagnosis; TI: therapeutic interventions; PP: patient perspective; IC: informed consent.

### Description of Studies and Analyses

The different variables analysed are given in Table 3.

**Table 3:** Summary of the clinical case reports

Author (year)	Gender / age	Oral manifestation / location	PRS AT	Associated symptoms	Diagnostic tests	Oral biopsy (yes/no) Biopsy results Location	Nonoral biopsy (yes/no) Results Location	Treatment	Oral lesion healing
Hérnandez et al (2008)	F / 64	Gingival tumor circumscribed to the oral cavity	1st NS	Fatigue Weakness Loss of appetite Sporadic pain in elbows, fingers and shoulders	Biopsy Blood analysis Thoracic radiograph	YES Granulomatous tissue, pseudoepitheliomatous hyperplasia, edematous connective tissue, vascular proliferation, chronic inflammatory infiltrate, plasmatic cells, microabscess, neutrophils and eosinophils Gingiva	NO	Prednisone 60 mg/day Cyclophosphamide 100 mg/day Methotrexate 7.5 mg/day/3 days per week	Yes
Hanisch et al (2017)	F / 72	Gingival hyperplasia	R 8 years	Glomerulonephritis Pulmonary emphysema Nephrosclerosis Maxillary sinusitis Raynaud phenomena Hypertonia Hearing loss	Biopsy ANCA assesment	YES Inflammation and parakeratosis with granulomatous infiltration of neutrophils Palatal gingiva	NO	Prednisone 10 mg/day Cyclosporine 150 mg/day Rituximab 375 mg/m^2^ / 1 dose weekly/4 weeks	Yes
Wang et al (2018)	F / 14	Facial paralysis	1st NS	Bilateral otalgia Hearing loss Fatigue Loss of appetite Gingival tumefaction	Blood analysis Thoracic radiograph CT scan	NO	YES Middle ear: granulomatous inflammation with necrosis and vasculitis; Lung: necrotizing and suppurative granulomatous inflammation with spots of necrotizing vasculitis	Methylprednisolone, rituximab, cyclophosphamide dose NS	Yes
Fonseca et al (2017)	F / 75	Gingival hyperplasia	1st NS	Dyspnoea Fever Diarrhea/vomiting Weight loss Sepsis CMV infection Chronic anemia CI	Biopsy Techniques Blood analysis Thoracic radiograph Immunohistochemistry techniques	YES Pseudoepitheliomatous epithelial proliferation, mixed inflammatory infiltrate, multinucleated giant cells, microabscess, eosinophils, blood extravasation, and no granulomas Gingiva	YES Lung: granulomatous reaction	Methylprednisolone 40 mg/day Azathioprine 50 mg/12 h	†
Sung et al (2015)	M / 57	Gingival hyperplasia	1st NS	Skin ulcers	Biopsy Blood analysis ANCA assessment Thoracic radiograph CT scan	YES Chronic inflammation with abcess, fibrinoid vasculitis, tissue granulation Gingiva	NO	Methotrexate 12.5 mg/day/1 time per week Prednisone 40 mg/day	Yes
Staines et al (2009)	F / 25	Gingival hyperplasia	R 2 years	NS	Biopsy Blood analysis CT scan MRI Pharynx endoscopy	YES Acute inflammatory infiltrate of neutrophils, extravasation of erythrocytes, multinucleated giant cells. Gingiva	NO	Rituximab 375 mg/m^2^/once per week/4 weeks Prednisolone 60 mg/day	Yes
Candau et al (2014)	F / 44	Granulomatous gingival hyperplasia	1st NS	Fever	Blood analysis ANCA assessment CT scan	YES Inflammation, necrosis, granulomas, multinucleated giant cells Gingiva	NO	Cyclophosphamide, prednisone Dose NS	Yes
Siar et al (2011)	F / 50	Gingival hyperplasia	1st NS	NS	Biopsy Thoracic radiograph	YES Stratified squamous and parakeratinised epithelium, pseudoepitheliomatous hyperplasia, granulomatous inflammatory tissue, neutrophils, eosinophils, abscess, plasmatic cells, lymphocytes, no necrotising vasculitis Gingiva	NO	Prednisolone 60 mg/day Cyclophosphamide 100 mg/day	Yes
Ruokonen et al (2009)	F / 51	Gingival hyperplasia	1st NS	Chronic fatigue Muscle and articular pain Abdominal pain Weight loss	Biopsy Blood analysis ANCA assessment Thoracic radiograph	YES Inflammatory infiltrate, lymphocytes, plasmatic cells, neutrophils, eosinophils multinucleated giant cells, necrosis Gingiva	NO	Methotrexate Dosage NS	NS
Carter et al (2008)	F / 56	Tongue necrosis and ulceration	Prog 19 days	Headache Sinus pain Difficulty to breath Productive cough and hemoptysis Bronchiectasis High blood pressure Bilateral hearing loss Lung edema RF Pulmonary hemorrhage	Blood analysis ANCA assessment Thoracic radiograph Nasal endoscopy	NO	NO	Cyclophosphamide Dosage NS	†
Xing et al (2011)	M / 6	Gingival hyperplasia Progressive periodontitis Oral ulcers (soft palate and oral mucosa in general)	1st NS	NS	Blood analysis ANCA assessment Thoracic radiograph Tuberculin test	YES Small blood vessels vasculitis, inflammatory cells infiltrate Palatal mucosa	NO	Imipenem and Cotrimoxazole (120 mg/day) Methotrexate (5 mg/Kg) Prednisolone (30 mg/day)	Yes
Ceylan et al (2013)	F / 37	Bilateral parotid tumefaction Skin fistulas	1st NS	Epixtasis Nasal obstruction Weight loss Cough Articular pain in knees, wrists and elbows Fatigue General discomfort	Blood analysis Rhinoscopy Tuberculin test Microbiological Culture FNA of parotid	YES Chronic and diffuse inflammation, necrosis, granulomas, Langerhans cells Parotid glands	YES Nasal cavity: mixed inflammatory infiltrate, histiocytes, diffuse granulomatosis, giant cell granulomas	Deltacortril 100 mg/day TMPSMX 80400 mg Cyclophosphamide 150 mg/day Methotrexate 10 mg/kg	Yes
Kenis et al (2013)	M / 60	Parotitis	1st NS	Fever Edema and mandibular pain Purulent secretion Facial paralysis	Blood analysis ANCA assessment Thoracic radiograph CT scan MRI	YES Massive necrosis of the parotid gland and soft tissue with chronic inflammation and acute granulomatous inflammation Parotid gland	YES Kidney: glomerulonephritis and fibrinoid necrosis	Intravenous corticosteroids, prednisone, cyclophosphamide Dosage NS	Yes
Green et al (2013)	M / 57	Parotitis	1st NS	Ear pain Purulent secretion Low fever Dyspnoea Weakness Back pain Arthralgia Leg edema	Blood analysis ANCA assessment MRI CT scan	YES Necrotic granulomas with no evidence of vasculitis Parotid gland	NO	Methylprednisolone 500 mg/day Cyclophosphamide 50 mg/day	NS
Kosalka et al (2014)	F / 57	Hard palate perforation	Pg 7 months	Thoracic pain Polyneuropathies Articular pain Hearing loss Perforations in bronchia and trachea	ANCA assessment CT scan	NO	YES Bronchi: inflammatory granuloma and necrosis	Cyclophosphamide (15 mg/kg) Methylprednisolone (1mg/kg) Prednisone (40 mg/day) Rituximab (375 mg/m2)	†
Reboll Ferrer et al (2010)	F / 53	Ulcer on the soft palate and tonsils	Pg 10 days	Odynophagia Hearing loss	Blood analysis ANCA assessment CT scan	YES Lymphoid follicular hyperplasia Palate, adenoid tissue	YES Mammary gland: chronic inflammatory infiltrate, giant cells, granulation tissue	Corticosteroids 1 mg/kg/day Prednisone 50 mg/24h Cyclophosphamide (50 mg/12 h)	Yes
Ujjawal et al (2016)	M / 50	Facial paralysis (NS unilateral and/or bilateral)	1st NS	Articular pain in knees and fingers Plantar edema	Blood analysis ANCA assessment Thoracic radiograph Neurological tests	NO	YES Kidney: fibrinoid necrosis, endocapillary proliferation, leukocytosis, glomerulonephritis	Methylprednisolone, 3 doses of 1g Cyclophosphamide 15 mg/kg/15 days Glucocorticoids (0,25 mg/kg) y azathioprine (260 mg/24hours)	Yes
Peters et al (2018)	F / 32	Unilateral facial paralysis	1st NS	Epistaxis Nasal congestion Recurrent bilateral otitis Progressive hearing loss	Biopsy Blood analysis ANCA assessment CT scan	NO	YES Nasopharynx, medium meatus, septum: inflammatory infiltrate of neutrophils, lymphocytes, plasmatic cells, histyocytes, eosinophils, small vessel necrosis vasculitis	Methotrexate 25 mg/week Prednisolone 60 mg/day Rituximab 1 g Azathioprine, dosage NS	NR
Gómez Torres et al (2013)	F / 33	Bilateral facial paralysis	Pg 2 months	Hearing loss Repetitive otitis Chronic dizziness Instability Nausea and vomiting	Blood analysis Thoracic radiograph CT scan MRI Tuberculin Test Culture Lumbar puncture	NO	NO	Methylprednisolone 1g Immunosuppressive	Yes
Heera R et al (2012)	M / 54	Gingival hyperplasia	1st NS	Discomfort	Blood analysis ANCA assessment Tuberculin test	YES Stratified squamous and parakeratinised epithelium, pseudoepitheliomatous hyperplasia, intraepithelial abscesses, inflammatory infiltrate of neutrophils, plasmatic cells, macrophages, dilatation and thickening of blood vessels Gingiva	NO	Prednisolone 20 mg/24h	NS
**CASE REPORT**	M / 25	Granulomatous lesions on attached gingiva Ulcer on tonsillar pillar Oroantral communication on the left maxilla Generalised periodontal disease	1st	Follicular lesions on skin Vasculitis lesions in legs Joints are affected	Blood analysis Urine test ANCA test Thorax test CTPET	YES Unspecific results Gingiva	YES Skin biopsy: vascultis process with thrombus associated to a neutrophil infiltrate and superficial necrotic tissue, dermal vascular damage with fibrinoid necrosis and polymorphonuclear infiltrate associated with intravascular thrombus	Intravenous methylprednisolone (20 mg/8h) Oral prednisone (60 mg/24h)	Yes

PRS: presentation; AT: average time until lesion emergence when it is recurrence and progression of the disease; NS: non-specified; F: female; M: male; CI: cardiac insufficiency; RF: renal failure; CT scan: computed tomography scan; MRI: magnetic resonance imaging; 1st: 1st manifestation; R: recurrence; Pg: disease progression; NOL: nonoral location; †: deceased; NR: non-recurrence; FNA: fine needle aspiration.

In all 20 papers analysed, only one case per paper was described. Of these 20 patients, 14 (70%) were women and 6 men (30%), with an average age was 47.4 years, and age at presentation ranging from 6 to 75 years. In terms of the location of oral and facial lesions, the most frequently affected area was gingiva (50%), followed by the facial nerve (20%), palate (15%), parotid gland (15%) and tongue (5%). The oral and facial lesions included 8 cases of gingival hyperplasia (45%), 1 gingival tumor (5%), 1 case of progressive periodontitis (5%), 4 with facial paralysis (20%), 3 ulcers on the oral mucosa (15%), 1 tongue necrosis (5%), 3 with parotitis (15%), 1 skin fistula (5%), and 1 palate perforation (5%) (Table 4). Of the 20 patients, 14 (70%) experienced oral lesions as a first manifestation of the disease, 4 (20%) as a progression of GPA and 2 (10%) as a recurrence of the disease. Concerning the lesions that appeared as a first manifestation, the most common location was the gingiva (40%), presenting as gingival hyperplasia, gingival tumor and progressive periodontitis. Facial paralysis and parotitis also occurred as a first manifestation. GPA recurrence manifested as gingival hyperplasia, with progression to ulceration and tongue necrosis (5%), palatal perforation (5%), palate ulcer (5%) and bilateral facial paralysis (5%).

**Table 4:** Possible oral manifestations and the frequency of each one in percent

Oral manifestation	Frequency (%)
Gingival hyperplasia	45
Gingival tumor	5
Progressive periodontitis	5
Facial paralysis	20
Ulcers on the oral mucosa	15
Tongue necrosis	5
Skin fistula	5
Palate perforation	5

The average time until re-appearance of the oral lesions when they were a recurrence of the disease was 3 years and 10 months when it was a progression of the disease.

The associated symptoms in the cases where the oral lesions presented as a first manifestation were: loss of appetite, sporadic pain, dyspnea, fever, diarrhea/vomiting, weight loss, sepsis, CMV infection, chronic fatigue, muscle and/or articular pain, abdominal pain, bilateral otalgia, hearing loss, epistaxis, nasal obstruction, cough, edema, mandibular pain and purulent secretion. Symptoms associated with GPA recurrence were glomerulonephritis, emphysema, nephrosclerosis, maxillary sinusitis, Raynaud syndrome, hypertonia, and hearing loss. Symptoms associated with the progression of the disease were headache, sinus pain, breathing difficulty, cough with purulent discharge or hemoptysis, bronchiectasis, hypertension, bilateral hearing loss, lung edema, renal insufficiency, pulmonary hemorrhage, thoracic and articular pain, polyneuropathy, hearing loss, tracheal and bronchial perforation, odynophagia, recurrent otitis, chronic dizziness, instability, nausea and vomiting.

Regarding histopathologic analysis, in 14/20 (70%), a biopsy was performed on a sample from an orofacial site (gingiva and/or parotid gland). Of these, 11/20 (55%) had the oral and/or facial lesion as the first manifestation, 2/20 (10%) had a recurrence, and 1/20 (5%) had a progression of the disease. Despite the fact that all the patients had oral manifestations, the biopsy was taken from a non-oral location in 3/20 patients (15%), e.g. lungs, middle ear, kidney, nasopharynx, medium meatus, and septum. In 2/20 patients (10%) biopsy was not performed; both of these were progressions of the disease. Also, 4/20 (20%) patients had a biopsy of other sites such as lungs, nasal cavity, kidney and mammary gland in addition to the oral biopsy; 3 of these 4 patients (3/20; 15%) had oral and/or facial lesions as a first manifestation and 1 (1/20; 5%) as a progression.

The histopathological findings of gingival hyperplastic lesions were: granulomatous areas, pseudoepitheliomatous hyperplasia, edematous connective tissue, vascular proliferation, chronic inflammatory infiltrate, plasmatic cells, microabscess, eosinophils, parakeratosis, neutrophils in granulomatous infiltrate, multinucleate giant cells and/or necrosis. Biopsy results were similar when the disease manifested as parotitis: chronic and diffuse inflammation, necrosis, granulomas and Langerhans cells. In the case of ulcers located in the soft palate, the biopsy showed lymphoid follicular hyperplasia.

The oral and/or facial lesions or symptoms present in patients who did not have an oral biopsy but did have one from other sites (3/20 patients) were facial paralysis and hard palate perforation. The patients who did not have any type of biopsy (2/20) had the following lesions or symptoms: ulcerations, tongue necrosis and facial paralysis.

The histopathological findings of pulmonary samples were: necrotising and suppurating granulomatous inflammation with spots of necrotising vasculitis as well as inflammatory granuloma. Samples from the nasal cavity showed mixed inflammatory infiltrate, collagenous necrosis, histiocytes, diffuse granulomatosis and giant cell granulomas. Kidney samples demonstrated glomerulonephritis and fibrinoid necrosis, endocapillary proliferation and leukocytosis. Mammary gland samples showed chronic inflammatory infiltrate, giant cells and granulation tissue. Granulomatous inflammation with focal necrosis and vasculitis were found in middle-ear samples. Lastly, the findings from the samples of nasopharynx, medium meatus and septum were: neutrophil inflammatory infiltrate, lymphocytes, plasmatic cells, histiocytes, eosinophils and necrotising vasculitis of small vessels.

Diagnostic tests performed on most of the 20 reported cases included a complete blood analysis, thoracic radiograph, ANCA assessment, magnetic resonance imaging (MRI) scan, computed tomography (CT) scan, tuberculin test and a neurologic test, among others.

Regarding the oral and facial lesion evolution after GPA treatment, in 13 patients (65%) the lesions were resolved, in 3 patients (15%) the resolution of the lesions was not specified, and in 3 cases (15%) the patient died. Two of these 3 deaths were due to the deterioration of the immune system associated with the progression of the disease . In one of the fatal cases, the oral lesion was the first manifestation of the disease. In one patient (5%), the presentation was a recurrence and the clinical manifestation was facial paralysis.

Of the 13 lesions (65%) that resolved after treatment, 9/13 (69.2%) presented as a first manifestation, 2/13 (15.4%) were a recurrence and 2/13 (15.4%) were a progression of the disease. Of these lesions, 1/13 (7.7%) was a gingival tumor, 7/13 (53.8%) were gingival hyperplasia, 3/13 (23.07%) were facial paralysis and 2/13 (15.38%) parotitis.

The three cases in which the resolution of the oral lesions was not specified demonstrated gingival hyperplasia and parotitis. In these 3 cases, the oral lesion was also the first manifestation of the disease. Finally, in the three fatal cases, the lesions were gingival hyperplasia, ulceration, tongue necrosis and perforation of the hard palate.

## Case Report

A 25-year-old male with no previous history of allergies or medical problems came to the dental office for the extraction of teeth with poor prognosis. The patient smoked 20 cigarettes per day, had generalised advanced periodontal disease and multiple septic areas in the oral cavity.

**Fig 1:**
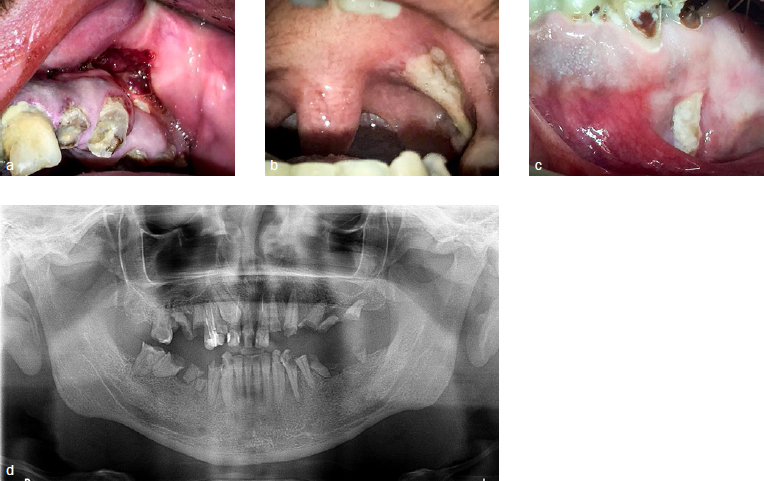
a) Ulcer with everted border in oral mucosa of the left maxilla; b) large ulcer in the left tonsillar pillar; c) mucosal fenestrations; d) initial panoramic radiograph.

**Fig 2:**
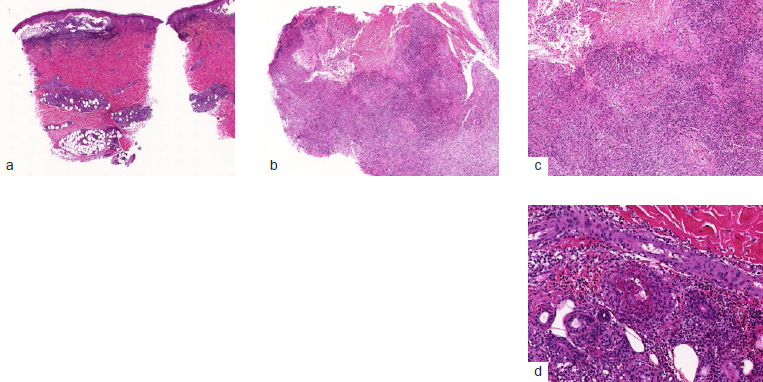
Histological images of the skin biopsy. a) Image of the skin biopsy taken with a magnifying lens. b) 50X detail of the skin biopsy. c) 100X detail of the skin biopsy; granulation tissue can be seen deep in the ulcer. d) 200X detail of the skin biopsy, showing a vessel with vasculitis.

Oral examination (performed in January 2018) showed granulomatous lesions, a large ulcer on the left tonsillar pillar and oro-antral communication in the left maxilla (Fig 1). Extraoral examination showed follicular lesions on the skin and vasculitis on legs and feet, as well as discrete articular involvment of wrist, knees and ankles. The patient was unaware of the time of appearance of these lesions and did not seem to give it any importance. On suspicion of a systemic illness, the patient was referred to the maxillofacial surgery service at Bellvitge Universitary Hospital, Barcelona, Spain. An incisional biopsy was performed on the granulomatous lesion localised on the alveolar mucosa of the left maxilla. The histological findings were inconclusive. A decision to carry out a skin biopsy was made with results showing: dermal vascular damage with fibrinoid necrosis and associated polymorphonuclear neutrophil infiltrate with the presence of intra-vessel thrombus (Fig 2).

**Fig 3:**
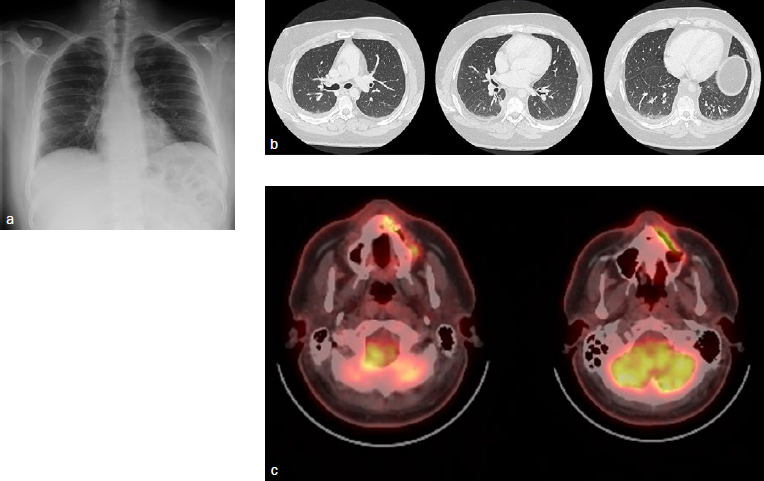
a) Thoracic radiograph: Discrete bilateral pleural effusion with subjacent parenchyma atelectasis. The remaining parenchyma has no significant alterations. b) Thoracic CT: Bilateral pleural effusion with areas of atelectasis in the subjacent parenchyma. Ganglia of unspecific size in the mediastinal territory with no signs of adenopathy. c) PET-CT of maxilla: hypermetabolism in left maxilla related with an increase of parts density and a fistulous tract in relation to an infectious-inflammatory process. Small submandibular laterocervical adenopathy (slightly contrast enhanced), related to a reactive-inflammatory process.

With the polyarticular signs and a blood analysis that showed normocytic anemia (Hb 106g/l, Ht 32.3%, MCV 84 fl [femtoliter]), thrombocytosis (879,000), leukocytosis (12,900), neutrophilia (9920), lymphopenia (1090), elevated CRP (246.7 mg/dl), the patient was referred to the rheumatologic service under the suspicion of an underlying autoimmune disease. Additionally, blood analysis (vasculitis profile), urine analysis, thoracic radiograph, CT scan and a positron emission tomography/computed tomography (PET-CT) (Fig 3) were performed.

Blood analysis results were positive for antiPR3 antibodies. Thoracic radiographs showed bilateral pleural occupation, elevated bilateral perihilar bronchovascular markings, subjacent parenchyma atelectasis and the remainder of the parenchyma without significant alterations.

The PET-CT revealed an inflammatory-infectious process in the left maxillary area and bilateral sub-mandibular adenopathy with a reactive-inflammatory appearance.

After assessing the test results, the conclusive diagnosis was Wegener’s granulomatosis, defined as a positive ANCA vasculitis with a antiPR3 pattern, with the following areas/organs affected: oral cavity (ulcers), joints (polyarthritis), muscles (myositis), kidneys (high blood pressure, proteinuria <1g, inflammatory sediment with microhematuria), lungs (bilateral pleurisy), skin and spleen (splenomegaly). Treatment started with intravenous methylprednisolone (20 mg/8 h/7 days) associated with the immune modulator rituximab (1g perfusion, 2 administrations). Treatment continued with oral prednisone (60 mg/24 h/30 days), decreasing the dose by 5 mg every two weeks, combined with the immunosuppressive azathioprine (50 mg/12 h). Additionally, an anti-hypertensive (enalapril 5 mg/12 h, bisoprolol 5 mg/24 h) an antiplatelet (acetylsalicylic acid 300 mg/day) and calcium (Ideos 50 mg/400 IU /12 h) are prescribed.

**Fig 4:**
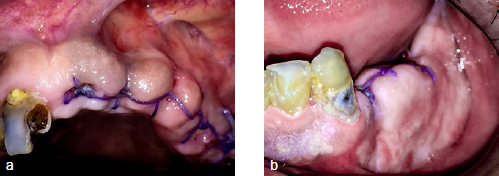
a) Check-up after dental treatment. Patient is stable. b) Healed mucosal fenestration.

The patient returned for dental treatment two months after the diagnosis was made, and was clinically stable. Dental treatment was performed after consulting with the rheumatology service and with antibiotic coverage and blood pressure monitoring. Multiple teeth were extracted and the patient decided to postpone the prosthetic rehabilitation treatment (Fig 4b).

**Fig 5:**
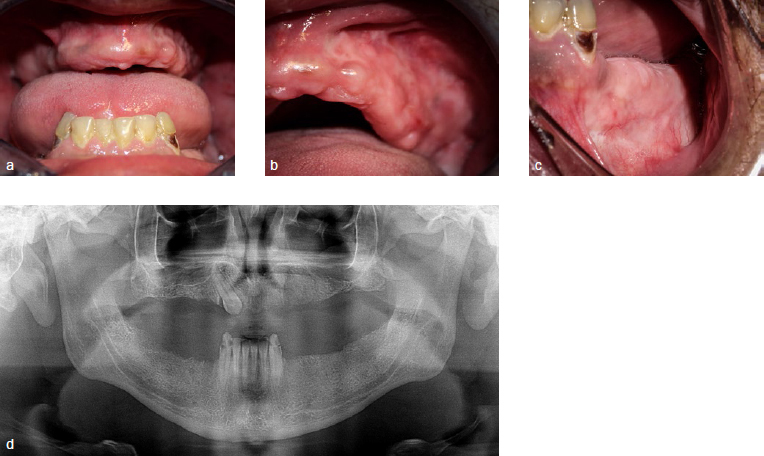
a) Follow up of the oral lesions one year after diagnosis. Patient is stable and pending prosthetic dental rehabilitation. b) Healed ulcer in maxilla. c) Patient scheduled to have conservative treatment in teeth of the mandible. d) Panoramic radiograph (December 2018).

At the present time, the patient is under treatment with prednisone (2.5 mg/day), azathioprine (50 mg/12h) and enalapril (5 mg/12h), continuing to be stable at the 1-year follow-up (Fig 5).

## Discussion

In the following, the diverse oral and facial manifestation of GPA are described using the clinical cases reported by various authors and these are compared with the data obtained in this review on the clinical case presented (Table 3).

Upper and lower airways as well as kidneys are the areas most frequently affected by GPA. According to the reviewed literature, oral lesions may occur in 6%-13% of the cases; however, only 2% develop as a first sign of the disease.^23^ Our patient had asymptomatic lung lesions and no kidney lesions but did have asymptomatic oral lesions as a first sign of GPA. Focusing on the oral manifestation, these include mucosa ulceration that can affect buccal and lingual mucosa, mouth floor, posterior pharynx, and tonsils, with a healing delay in these areas.^1^,^36^ As previously mentioned, GPA has no gender preference, but the data found in this review indicate that oral and facial manifestations seem to be more frequent in women (70% women vs 30% men). Nevertheless, our patient was male and had multiple oral lesions that were not the main motive for consultation, since he came for a checkup and to resolve his dental problems.

Authors such as McKinney et al^23^ state that when the first manifestation of GPA is in the oral cavity, it is usually as gingival tumefaction and unspecific ulceration. These observations concur with Eufinger et al,^6^ who reviewed 85 clinical case reports published between 1930 and 1990, finding that 72.7% of the oral lesions were oral ulcers and 27.3% were gingival hyperplasia. In contrast, the results of the present review of 20 clinical case reports found the most frequent location of the primary oral lesions produced by GPA to be the gingiva (50%) in the form of gingival hyperplasia (45%), gingival tumors and periodontitis, with oral ulcers accounting for only 15% of the lesions. Our patient had granulomatous lesions in the attached gingiva, an ulcer in the tonsillar pillar and generalised periodontal disease. However, other types of lesions can also be present as a first manifestation of the disease, although less frequently, such as parotitis and facial paralysis. Almouhawis et al^1^ reported that other areas of the oral cavity and craniofacial region can also be affected, such as the tongue, alveolar bone, palate, lips, teeth, oropharynx, salivary glands, cranial nerves and cranial bones.

Hernández et al^16^ and Staines et al^33^ stated that when the disease affects the gingiva, it does so in diverse forms: gingival enlargement, strawberry gingivitis, gingival erythema, petechiae, bleeding, ulceration and necrosis. This agrees with the results of the present review and with the clinical signs of our patient. Gingival lesions can be a first manifestation or a recurrence of GPA. When the lesions are a recurrence, they often appear in areas where a dental extraction has been performed. According to the present review, they show as hyperplasia. Furthermore, this study affirms that GPA can delay healing after a tooth extraction, as in the case presented by Eufinger et al.^6^ According to the data obtained in this review, 3 years is the average time until re-appearance of the oral lesions when it is a recurrence, and 10 months when it is a progression of the disease. In our case, after a 1-year follow up, the lesions have not recurred after tooth extractions.

Hanisch et al^14^ reported a case where GPA recurrence manifested in the oral cavity as a gingival hyperplasia (the initial diagnosis in 2007 did not show an oral lesion as the first manifestation of the disease) that did not respond to periodontal treatment. Those authors performed a biopsy which showed histopathologic signs characteristic of GPA. A blood test to determine the presence of ANCA was positive, confirming disease recurrence. Ruokonen et al^31^ reported a clinical case in which strawberry gingivitis was the first manifestation of GPA. Diagnosis was established after performing an incisional biopsy of the lesion and laboratory tests. Later, Siar et al^32^ described a similar case, but these authors also carried out a thoracic radiograph to confirm GPA diagnosis. In the review by Eufinger et al,^6^ 42% of the patients examined had gingival hyperplasia as a first manifestation, making this percentage similar to the one obtained in the present review (40%).

On the other hand, Hernández et al^16^ described the first case where Wegener’s granulomatosis was manifested as a circumscribed tumor in the oral cavity. The lesion was a firm mass, located in the attached and marginal gingiva of the central incisors, with a diameter of 2 cm, round, red in colour, with well-defined limits and with a distinctive granular and speckled surface. Both Hernández et al^16^ and Carter et al^3^ stated that the presence of gingivitis with a dark red colour and strawberry-like appearance with a granular surface affecting one or several gingival papilla can be considered specific of GPA, and is one of the most common oral manifestations of the disease.

The clinical cases in this review confirm that when GPA is manifested in the gingiva, the most common characteristics are slightly painful, edematous, hyperplasic and erythematous gingivae with petechiae especially affecting the anterior and interpapillary area of the maxilla; bleeding is also frequent in the area. GPA can impact one or both dental arches and the signs and symptoms do not resolve after periodontal treatment.^2^,^9^,^36^ Histological features usually include pseudoepitheliomatous proliferation and an intense mixed inflammatory infiltrate with multinucleated giant cells, microabscess, eosynophils and blood extravasation to the connective tissue, fibrinoid vasculitis and granulation tissue.^9^,^36^

Although infrequent, GPA can appear in children as progressive periodontitis. Xing et al^40^ reported a case of a 6-year-old boy who had abnormal tooth loss, progressive periodontitis and painful oral ulcers. These symptoms were the first manifestation of GPA.

In relation to tongue involvement, GPA can manifest as ulceration and necrosis. Tongue necrosis as a GPA oral manifestation is infrequent and in the few cases published in the literature, like the one described by Carter et al,^3^ it occurs when the disease is in an advanced state and afffects other organs and systems as well. This is corroborated in the review by Eufinger et al,^6^ which found that oral ulcers occurred in 53% of patients, but as a result of the disease progression. This differs from the present review, in which only 10% of the assessed patients had oral ulcers and always as a progression of the disease. Our patient had ulcerous lesions on the left tonsillar pillar, which was the first manifestation of GPA.

According to Almouhawis et al,^1^ when the alveolar bone is affected, GPA produces osteomyelitis and bone resorption, leading to tooth mobility and loss. Afflicted lips may experience sensitivity loss, nodular masses, loss of the vermillion border, desquamation and lip edema. However, no clinical case report was found in the literature in the last ten years that describes these lesions.

Almouhawis et al^1^ state that when the palate is affected, the signs may include ulceration, osteonecrosis and oroantral fistula, although they are claimed to be unusual and not a first manifestation of GPA. Taking into consideration the results of this review, we disagree because Reboll-Ferrer et al^29^ described a case where the soft palate was affected by an ulcer; this was the first manifestation of GPA, along with other clinical findings such as thoracic abscesses. Furthermore, Kosalka et al^20^ reported a patient that had a perforation on the hard palate due to GPA. Moreover, perforations were also seen in the bronchiae; these GPA oral manifestations were present in disease progression. Our patient had an oro-antral communication in the left maxilla that healed spontaneously when the disease was controlled. As the evolution time of the core disease in our patient was unknown, there was no way to determine whether the cause of tooth loss in that area was GPA or not.

According to the literature,^20^ affliction of the major salivary glands as a first manifestation of GPA is uncommon. However, the present review showed that in 15% of the assessed cases, the parotid gland was affected. This, too, was a first manifestation of the disease in all of the cases. GPA affecting the parotid gland was first described by Fahey et al.^7^ GPA can enlarge the parotid gland and produce unilateral or bilateral pain, as in the case described by Ceylan et al.^5^ On the other hand, Kenis et al^19^ described a case where the first manifestation of GPA was in the parotid gland and the signs and symptoms were pain, edema and purulent exudate with numerous calcifications. Green et al^13^ reported a similar case in which the parotid gland was affected, occurred as a first manifestation of GPA, and was associated with ear pain and purulent secretion.

Lastly, the central nervous system is affected in a significant proportion of patients with GPA, manifesting as multiplex mononeuritis due to vasculitis and infiltration of the granulomatous lesions (10%-45%). According to the consulted literature, the cranial nerves affected most frequently are numbers II, VI and VII, while the least affected are numbers IX, X and XII. According to some authors,^38^ cranial neuropathy is uncommon as a first manifestation of GPA. It usually presents as a facial paralysis, unilateral or bilateral, vocal cord paralysis or hearing loss.^28^,^39^ However, of the 20 cases analysed in the present review, the facial nerve was affected in 4 (20%) of them; in 3 of these, it was a first manifestation of GPA. Only the case reported by Gómez-Torres et al^11^ described facial paralysis as a recurrence of the disease. It was bilateral and accompanied by hearing loss, but after treating the disease, the symptoms improved.

An important limitation of this review is that all the articles assessed only one case per paper, and some of the variables examined were not reported in some articles. As an example of this, the average time until re-appearance was not reported in 11 of the 20 studied cases and in 3 of these there is no data about the associated symptoms of the disease.

## Conclusion

GPA is a multisystem disorder associated with considerable mortality and morbidity if left untreated. This is why it is important to recognise the signs and symptoms associated with the disease, due to the fact that an early diagnosis can remarkably improve the prognosis.

The first manifestation of the disease can arise in the oral cavity, especially in form of strawberry gingivitis and gingival hyperplasia. In approximately 70% of the cases, these lesions usually resolve favorably after treatment.

If a patient is suspected to have GPA at the dental visit, a corresponding blood test must be requested and referral to a rheumatology and/or internal medicine clinic must be made to rule out the presence of this disease or to achieve an early diagnosis and treatment. For the dental management of GPA, the same recommendations pertaining to patients under immunosuppressive therapy must be followed.

## References

[ref1] Almouhawis HA, Leao JC, Fedele S, Porte SR (2013). Wegener’s granulomatosis: a review of clinical features and an update in diagnosis and treatment. J Oral Pathol Med.

[ref2] Candau A, Valenzuela B, Dean A, Alamillos FJ (2014). Wegener’s granulomatosis with oral mucosal involvement as first manifestation. Acta Otorrinolaringol Esp.

[ref3] Carter LM, Brizman E (2008). Lingual infarction in Wegener’s Granulomatosis: a case report and review of the literature. Head Face Med.

[ref4] Chang SY, Keogh KA, Lewis JE, Ryu JH, Cornell LD, Garrity JA, Yi ES (2013). IgG4-positive plasma cells in granulomatosis with polyangiitis (Wegener’s): a clinicopathologic and immunohistochemical study on 43 granulomatosis with polyangiitis and 20 control cases. Hum Pathol.

[ref5] Ceylan A, Asal K, Celenk F, Koybasioglu A (2013). Parotid gland involvement as a presenting feature of Wegener’s granulomatosis. Singapore Med J.

[ref6] Eufinger H, Machtens E, Akuamoa-Boateng E (1992). Oral manifestations of Wegener’s granulomatosis. Review of the literature and report of a case. Int J Oral Maxillofac Surg.

[ref7] Fahey JL, Leonard E, Churg J, Godman G (1954). Wegener Granulomatosis. Am J Med.

[ref8] Falk RJ, Gross WL, Guillevin L, Hoffman G, Jayne DR, Jennette JC (2011). Granulomatosis with polyangiitis (Wegener‘s): an alternative name for Wegener‘s granulomatosis. J Am Soc Nephrol.

[ref9] Fonseca FP, Benites BM, Ferrari A, Sachetto Z, de Campos GV, de Almeida OP (2017). Gingival granulomatosis with polyangiitis (Wegener’s granulomatosis) as a primary manifestation of the disease. Aust Dent J.

[ref10] Godman GC, Churg J (1954). Wegener’s granulomatosis: pathology and review of the literature. AMA Arch Pathol.

[ref11] Gómez-Torres A, Tirado Zamora I, Abrante Jimenez A, Esteban Ortega F (2013). Wegener’s granulomatosis causing bilateral facial paralysis and deafness. Acta Otorrinolaringol Esp.

[ref12] Greco A, Marinelli C, Fusconi M, Macri GF, Gallo A, De Virgilio A (2016). Clinic manifestations in granulomatosis with polyangiitis. Int J Immunopathol Pharmacol.

[ref13] Green I, Szyper-Kravitz M, Shoenfeld Y (2013). Parotitis as the presenting symptom of Wegener’s granulomatosis: case report and meta-analysis. Isr Med Assoc J.

[ref14] Hanisch M, Frohlich LF, Kleinheinz J (2017). Gingival hyperplasia as first sign of recurrence of granulomatosis with polyangiitis (Wegener’s granulomatosis): case report and review of the literature. BMC Oral Health.

[ref15] Heera R, Choudhary K, Beena VT, Simon R (2012). Strawberry gingivitis: A diagnostic feature of gingival Wegener’s granulomatosis. Dent Res J (Isfahan).

[ref16] Hernández G, Serrano C, Porras L, Lopez-Pintor R, Rubio L, Yanes J (2008). Strawberry-like gingival tumor as the first clinical sign of Wegener’s granulomatosis. J Periodontol.

[ref17] Holle JU, Gross WL, Holl-Ulrich K, Ambrosch P, Noelle B, Both M (2010). Prospective long-term follow-up of patients with localised Wegener’s granulomatosis: does it occur as persistent disease stage?. Ann Rheum Dis.

[ref18] Jennette JC, Falk RJ, Bacon PA, Basu N, Cid MC, Ferrario F (2013). 2012 revised International Chapel Hill Consensus Conference Nomenclature of Vasculitides. Arthritis Rheum.

[ref19] Kenis I, Zahavi T, Korzets Z (2013). Parotid gland involvement as initial presentation of Wegener’s granulomatosis: a diagnostic pitfall. Isr Med Assoc J.

[ref20] Kosalka J, Bazan-Socha S, Zugaj A, Ignacak M, Zuk J, Sokołowska B (2014). Granulomatosis with polyangiitis (Wegener’s granulomatosis) with hard palate and bronchial perforations treated with rituximab – a case report. Pneumonol Alergol Pol.

[ref21] Leavitt RY, Fauci AS, Bloch DA, Michel BA, Hunder GG, Arend WP (1990). The American College of Rheumatology 1990 criteria for the classification of Wegener’s granulomatosis. Arthritis Rheum.

[ref22] Lima AM, Torraca PF, Rocha SP, Santiago CMR, Ferraz FHRP (2015). Granulomatosis with polyangiitis, a new nomenclature for Wegener’s granulomatosis – case report. An Bras Dermatol.

[ref23] McKinney EF, Willcocks LC, Broecker V, Smith KG (2014). The immunopathology of ANCA-associated vasculitis. Semin Immunopathol.

[ref24] Moher D, Liberati A, Tetzlaff J, Altman DG, PRISMA Group (2009). Preferred reporting items for systematic reviews and meta-analyses: The PRISMA statement. Ann Internal Med.

[ref25] Morgan MD, Day CJ, Piper KP, Khan N, Harper L, Moss PA (2010). Patients with Wegener’s granulomatosis demonstrate a relative deficiency and functional impairment of T-regulatory cells. Immunology.

[ref26] Pajor AM, Kwiatkowska S, Kroczynska-Bednarek J, Piotrowski WJ (2015). Acute laryngeal dyspnea as first presentation of granulomatosis with polyangiitis. Pneumonol Alergol.

[ref27] Ponniah I, Shaheen A, Shankar KA, Kumaran MG (2005). Wegener’s granulomatosis: the current understanding. Oral Surg Oral Med Oral Pathol Oral Radiol Endod.

[ref28] Peters JE, Gupta V, Saeed IT, Offiah C, Jawad ASM (2018). Severe localised granulomatosis with polyangiitis (Wegener’s granulomatosis) manifesting with extensive cranial nerve palsies and cranial diabetes insipidus: a case report and literature review. BMC Neurol.

[ref29] Reboll-Ferrer RM, Zapater-Latorre E, Calabuig-Crespo C, Basterra-Alegria J (2010). Wegener’s granulomatosis: description of a case with oral manifestation. Med Oral Patol Oral Cir Bucal.

[ref30] Riley DS, Barber MS, Kienle GS, Aronson JK, von Schoen-Angerer T, Tugwell P (2017). CARE guidelines for case reports: explanation and elaboration document. J Clin Epidemiol.

[ref31] Ruokonen H, Helve T, Arola J, Hietanen J, Lindqvist C, Hagstrom J (2009). “Strawberry like” gingivitis being the first sign of Wegener’s granulomatosis. Eur J Intern Med.

[ref32] Siar CH, Yeo KB, Nakano K, Nagatsuka H, Tsujigiwa H, Tomida M (2011). Strawberry gingivitis as the first presenting sign of Wegener’s granulomatosis: report of a case. Eur J Med Res.

[ref33] Staines KS, Higgins B (2009). Recurrence of Wegener’s granulomatosis with de novo intraoral presentation treated successfully with rituximab. Oral Surg Oral Med Oral Pathol Oral Radiol Endod.

[ref34] Stewart C, Cohen D, Bhattacharyya I, Scheitler L, Riley S, Calamia K (2007). Oral manifestations of Wegener’s granulomatosis: a report of three cases and a literature review. J Am Dent Assoc.

[ref35] Stone JH (2013). Wegener’s Granulomatosis Etanercept Trial Research Group. Limited versus severe Wegener’s granulomatosis: baseline data on patients in the Wegener’s granulomatosis etanercept trial. Arthritis Rheum.

[ref36] Sung IY, Kim YM, Cho YC, Son JH (2015). Role of gingival manifestation in diagnosis of granulomatosis with polyangiitis (Wegener’s granulomatosis). J Periodontal Implant Sci.

[ref37] Tomasson G, Davis JC, Hoffman GS, McCune WJ, Specks U, Spiera R (2014). Brief report: The value of a patient global assessment of disease activity in granulomatosis with polyangiitis (Wegener’s). Arthritis Rheumatol.

[ref38] Ujjawal R, Koushik P, Ajay P, Subrata C A case of Wegener’s granulomatosis presenting with unilateral facial nerve palsy. Case Rep Med.

[ref39] Wang JC, Leader BA, Crane RA, Koch BL, Smith MM, Ishman SL (2018). Granulomatosis with polyangiitis presenting as facial nerve palsy in a teenager. Int J Pediatr Otorhinolaryngol.

[ref40] Xing X, Zhang T, Wang X (2011). Pediatric Wegener’s granulomatosis with oral ulcers and progressive periodontitis: a case report. Oral Surg Oral Med Oral Pathol Oral Radiol Endod.

